# Treatment of Pulmonary Consumption

**Published:** 1870-12

**Authors:** 


Treatment of Pulmonary Consumption.—Dr. C. J. B. Williams of London sums up, in the *Lancet*, the result of his forty years of observation on this subject somewhat as follows:

When he began practice two years was regarded as the limit of life of the consumptive.

Ten years showed a marked improvement in results of treatment, mainly from the use of alterative tonics, such as iodide of potassium, with sarsaparilla or other vegetable tonics.

At this time a good article of cod liver oil began to be procurable, and he has no hesitancy in saying that this agent has done more for the consumptive than all other means put together. Its usefulness and efficiency have gone on increasing in proportion to the greater facility for obtaining it pure, and to the improvement in the manner of giving it in combination with various tonics, and in conjunction with certain rules of diet and regimen.

As a result, during the forty years, the average life of the consumptive, under proper treatment, has increased from two to eight years.

Regarding consumption as a disease of degeneration and decay, his treatment is, of course, for the most part sustaining and invigorating, consisting of such means as rich food, tonics, stimulants, fresh air, and the like. But as many cases originate in inflammation, and are aggravated and kept up by it, a mild antiphlogistic treatment is at certain stages often called for, such as salines, at times antimony, catsplasms, leeches, and blisters. He thinks there is at present a tendency to ignore too much this indication.

The tonics he makes use of mainly are quinine, salacine, and iron; but the pure bitter tonics are often found very beneficial, as also mineral acids, which he almost invariably gives in conjunction with the cod liver oil.

But the latter agent is most of all important.

This he rarely gives in doses greater than a half-ounce three times a day, and he interdicts, while it is being administered, all other fatty substancces.

When cod liver oil cannot be taken, he falls back upon the old treatment, with iodide of potassium, nitric acid, and a bitter tonic, adding to each dose a drachm or two of pure glycerine.

The hypophosphites of soda and lime have in his hands proved decidedly beneficial in certain cases.

In cases where the larynx or trachea are effected, or there is convulsive cough, or offensive expectoration, inhalations are quite valuable.

He regards the inhaling instruments of little use, but prefers a quart jug of hot water into which the medicine is dropped. A napkin rolled into the shape of a tube is placed over the nozzle, and from this the patient inhales the steam impregnated with the medicine. This to be used a few minutes each night.

The drugs proven to be most valuable for this means of exhibition are creosote or carbolic acid, iodine, chloroform, oil of turpentine, and juice or extract of hemlock; a few drops of one or each of several being dropped into the water when used.

Under careful treatment the life of the consumptive may be prolonged for many years, and in not a few cases he may be cured.—*Richmond and Louisville Medical Journal*

